# A Proline-Based Neuraminidase Inhibitor: DFT Studies on the Zwitterion Conformation, Stability and Formation

**DOI:** 10.3390/ijms10093918

**Published:** 2009-09-07

**Authors:** Zhi-Wei Yang, Xiao-Min Wu, Li-Jun Zhou, Gang Yang

**Affiliations:** 1 Key Laboratory of Forest Plant Ecology, Ministry of Education, Northeast Forestry University, Harbin 150040, China; E-Mails:yzws-123@163.com (Z.-W.Y.);dolphin1009@sina.com (X.-M.W.);zlj_1008@yahoo.com.cn (L.-J.Z.); 2 Institute of Theoretical Chemistry, Shandong University, Jinan 250100, China

**Keywords:** density functional calculations, neuraminidase inhibitor, relative stability, water, zwitterions

## Abstract

The designs of potent neuraminidase (NA) inhibitors are an efficient way to deal with the recent “2009 H1N1” influenza epidemic. In this work, density functional calculations were employed to study the conformation, stability and formation of the zwitterions of 5-[(1*R*,2*S*)-1-(acetylamino)-2-methoxy-2-methylpentyl]-4-[(1*Z*)-1-propenyl]-(4*S*,5*R*)-d-proline (BL), a proline-based NA inhibitor. Compared to proline, the zwitterion stability of BL is enhanced by 1.76 kcal mol^−1^ due to the introduction of functional groups. However, the zwitterion of BL will not represent a local minimum on the potential energy surface until the number of water molecules increases up to two (n = 2). With the addition of two and three water molecules, the energy differences between the zwitterions and corresponding canonical isomers were calculated at 3.13 and −1.54 kcal mol^−1^, respectively. The zwitterions of BL are mainly stabilized by the H-bonds with the water molecules, especially in the case of three water molecules where the carboxyl-O atoms are largely coordination-saturated by three H-bonds of medium strengths, causing the zwitterion stability even superior to the canonical isomer. With the presence of two and three water molecules, the energy barriers for the conversion processes from the canonical isomers to the zwitterions are equal to 4.96 and 3.13 kcal mol^−1^, respectively. It indicated that the zwitterion formation is facile to take place with addition of two molecules and further facilitated by more water molecules. Besides, the zwitterion formation of BL is finished in a single step, different from other NA inhibitors. Owing to the above advantages, BL is a good NA inhibitor candidate and more attention should be paid to explorations of BL-based drugs.

## 1. Introduction

Influenza A (H1N1) is a subtype of influenza virus A and the most common cause of influenza in humans. The recent “2009 H1N1” influenza epidemic has put the whole World on alert [1,2]. Presently there are only restricted antiviral agents that can be considered to prevent and treat the influenza [3–6]. In addition, WHO was just informed that the H1N1 viruses are resistant to the most widely used antiviral drug—oseltamivir (known as Tamiflu^®^) [7,8]. Accordingly, the development of new generation anti-influenza drugs is of special urgency and significance [3–6].

**Scheme 1.**
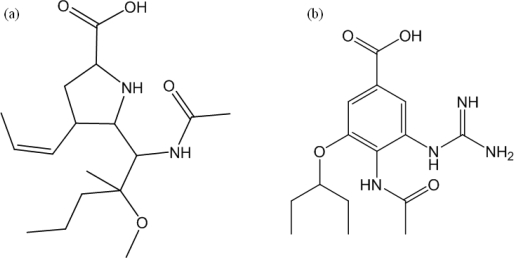
Structures of: (a) 5-[(1*R*,2*S*)-1-(acetylamino)-2-methoxy-2-methylpentyl]-4-[(1*Z*)-1-propenyl]-(4*S*,5*R*)-d-proline (BL) and (b): 4-(*N*-acetylamino)-5-guanidino-3-(3-pentyloxy) benzoic acid (BA).

It was found that a major surface glycoprotein of influenza, neuraminidase (NA), is an effective target to control the influenza virus. The active-site residues of different NA subtypes are highly conserved [4,9–12]. Inhibition of the NA proteins will delay the release of virions and thus allow sufficient time for host immune systems to remove the infected viruses [13–15]. Recently, 5-[(1*R*,2*S*)-1-(acetylamino)-2-methoxy-2-methylpentyl]-4-[(1*Z*)-1-propenyl)-(4*S*,5*R*)]-d-proline (Scheme 1) has been successfully designed and shown potential application as an anti-influenza drug [16–18].

The NA inhibitors usually have two distinct isomers; *i.e.,* neutral and zwitterionic. The neutral isomer of 4-(*N*-acetylamino)-5-guanidino-3-(3-pentyloxy)benzoic acid (BA) is shown in Scheme 1b. The transfer of the carboxyl H atom to the guanidine group forms the zwitterionic isomer. In the previous work [19], density functional calculations were used to study the proton transfer from the neutral to the zwitterionic isomers of BA, in the presence of water solvent. Among the known NA inhibitors, BL is special in that it was designed on basis of the proline amino acid (Scheme 1a). Accordingly, the direct proton transfer from the carboxyl group to the amine group can be realized, which may facilitate the zwitterion formation.

As is known to us, the electric fields of amino acid zwitterions are the driving forces that determine the functions of biomolecules [20,21]; in addition, the zwitterions are generally acknowledged as the active form of NA inhibitors [22–26]. Various strategies have been used to stabilize the zwitterions [20,21,27–42], including the hydration by water molecules [37–42]. In this work, density functional calculations were used to study the conformation, stability and formation of the zwitterionic isomer of BL with the gradual addition of water molecules. Besides, the relative stabilities of the BL and proline zwitterions in the absence of water were evaluated. The zwitterion of proline approximates 15.0 kcal mol^−1^ higher in energy than the canonical form [20]. The effects of the functional groups in BL on the zwitterion stabilities were obtained by the above evaluations. The canonical isomers of BL were studied as well, which were then compared with the zwitterions. As the assimilation of drugs takes place in aqueous solutions, this work will greatly facilitate the understanding towards the efficient designs of NA inhibitors.

## 2. Results and Discussion

### 2.1. Effects of Functional Groups on Zwitterion Stabilities

**Figure 1.**
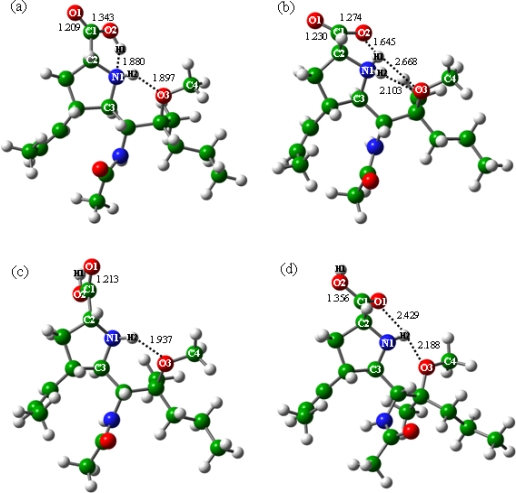
Structures of BL isomers in the absence of water: (a) BLa, (b) BLb, (c) BLc and (d) BLd. The N1-H1 distance in the zwitterionic isomer (BLb) was fixed at 1.030 Å.

**Figure 2.**
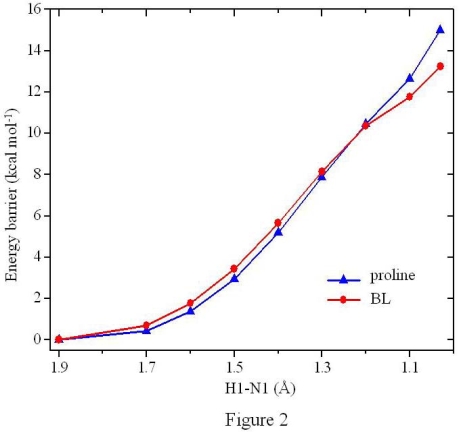
The energy profiles for the conversion processes from the canonical isomers to the zwitterions.

The geometry optimization process indicated that the zwitterion of BL does not exist independently in gas phase and will spontaneously convert to the canonical isomer (see BLa in Figure 1a). In BLa, the C1-O2 and C1-O1 distances are equal to 1.343 and 1.209 Ǻ, and the two H-bonds of N1-H1 and O3-H2 are equal to 1.880 and 1.897 Ǻ, respectively. Through the gradual decrease of the N1-H1 distance, the energy profile for the conversion process from the canonical isomer to zwitterion was obtained and plotted in Figure 2. It was found that the energies increase monotonously with the decrease of the N1-H1 distance. The zwitterion was evaluated with the N1-H1 distance of 1.030 Ǻ [21,35], see BLb in Figure 1b. In BLb, the H1 atom is bonded to the amine-N1 atom instead of the carboxyl-O2 atom in BLa. Both of the carboxyl C-O distances approximate 1.250 Ǻ, and the O2-H1 H-bond was optimized at 1.645 Ǻ. The O3 atom forms H-bonds with both amine-H atoms, with the O3-H1 and O3-H2 distances of 2.668 and 2.103 Ǻ, respectively. The presence of the aether-O3 atom may probably enhance the relative stability of the zwitterion, and its energy was estimated 13.25 kcal mol^−1^ larger than that of the corresponding canonical isomer (BLa).

The zwitterion of proline is not a local minimum on the potential energy surface (PES), which is consistent with the previous experimental and computational results [43–46]. It also agrees with the above analysis of BL isomers and the energy-profile calculations shown in Figure 2. The zwitterion of proline was estimated 15.01 kcal mol^−1^ higher in energy than its corresponding canonical isomer, in excellent agreement with the previous evaluation of about 15 kcal mol^−1^ [20].

The introduction of the functional groups to proline to form BL increases the relative stability of the zwitterions by 1.76 kcal mol^−1^; however, the functional groups are not enough to induce the zwitterion as a local minimum on the PES. After careful geometric analysis, it was found the main difference between the zwitterionic and canonical isomers of BL lies in the ether-O3 atom, which forms two H-bonds with the protonated amine group in the zwitterion (BLb) rather than one in the canonical isomer (BLa). It thus stabilizes somewhat the zwitterion of BL compared with proline where no functional groups are present [36].

### 2.2. Conformational Analysis of BL

As the assimilation of NA drugs takes place in aqueous solutions and the hydration by water molecules can stabilize the zwitterions [37–42], the introduction of water molecules to the BL structures were studied here. The BLx structures with addition of water molecules are defined to BLxn, where n refers to the number of water molecules. For example, the BLb2 represents the BLb isomer with addition of two water molecules (n = 2). The construction of the BLxn structures was based on the BLx(n-1) structures.

#### 2.2.1. In the absence of water (n = 0)

In addition to the BLa structure discussed above, another two isomers exist for the BL molecule in the absence of water, see BLc and BLd in Figure 1. The main geometric differences among the three isomers (BLa, BLc and BLd) are the orientations of the carboxyl-H1 atoms. There is one intra-carboxyl H-bond in each of BLc and BLd, with the O2-H1 and O1-H1 distances of 2.266 and 2.281 Ǻ; however, the direction of the H1 atom towards the amine N1 atom in BLa causes the disappearance of the intra-carboxyl H-bond. Although the formation of the intra-carboxyl H-bond, the carboxyl-amine H-bond still exists in BLd, with the O1-H2 distance of 2.429 Ǻ. The aether-O3 atoms of all the three isomers form one hydrogen bond with the amine-H2 atoms, and the distances were optimized to be 1.897 (BLa), 1.937 (BLc) and 2.188 (BLd) Ǻ, respectively.

The relative stabilities of the three BL isomers increase in the order BLc < BLd (−2.31 kcal mol^−1^) < BLa (−4.29 kcal mol^−1^). The values in parentheses are the relative energies with the total energy of BLc set as the benchmark. The BLa structure is of the lowest energy; in addition, it is the isomer that can transfer the carboxyl-H1 atom to the amine group and form the zwitterion. Accordingly, only the BLa and BLb isomers will be considered in the following discussions.

#### 2.2.2. With addition of water molecules

**Figure 3.**
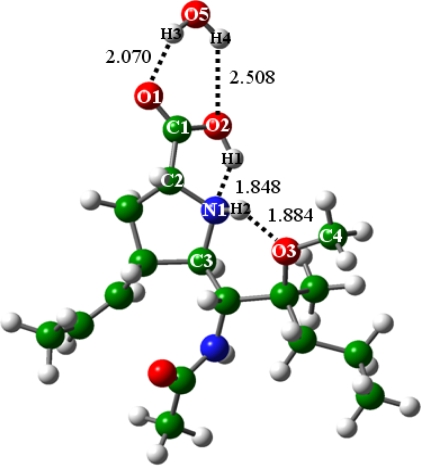
Structure of BLa with addition of one water molecule (BLa1).

The addition of one water molecule exerts slight influences on the geometries of BL isomers [37,47,48], see Figure 3. The zwitterion (BLb1) does not represent a local minimum on the PES, similar to the situation in the absence of water (n = 0). It is also consistent with the conformational searches of the proline molecule [48]. The water molecule forms two H-bonds with the BLa structure, with the exact distances given in Figure 3.

**Figure 4.**
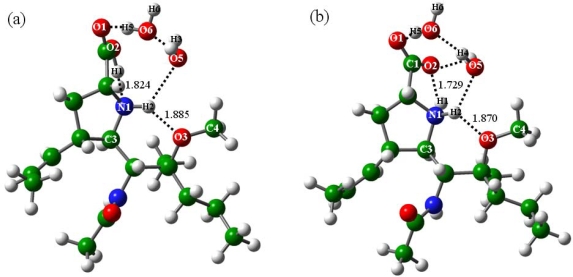
Structures of the BL isomers with addition of two water molecules: (a) BLa2, (b) BLb2.

With the presence of two water molecules (n = 2), the zwitterion of BL (BLb2) is rendered geometrically stable. The structures of BLa2 and BLb2 are shown in Figure 4. In BLa2, the C1-O2 and C1-O1 bond distances are equal to 1.331 and 1.221 Ǻ, and the N1-H1 and O3-H2 H-bonds are equal to 1.824 and 1.885 Ǻ, respectively. In BLb2, the C1-O2 and C1-O1 bond distances were optimized at 1.265 and 1.246 Ǻ, and the O2-H1 and O3-H2 H-bonds at 1.729 and 1.870 Ǻ, respectively. Besides the intra-BL H-bonds, the two water molecules form two and three H-bonds with the BL molecules in the BL2a and BL2b structures, respectively, with the detailed information given in Figure 4. It was found that except the bonding situations of the H1 atoms, the BL2a and BL2b geometries resemble each other. The zwitterion (BL2b) is probably stabilized mainly by the O5-H2 H-bond; in addition, the carboxyl-O atoms of BLb2 are somewhat coordination-saturated by the two H-bonds formed with the water molecules, which further stabilizes the zwitterionic structure. BL2b is higher in energy than BL2a, and the energy difference was calculated to be 3.13 kcal mol^−1^. Compared with glycine studied by Jensen *et al.* [37], the two water molecules improve the zwitterion stability more obviously in the case of BL; *i.e.,* the proline molecule with addition of functional groups.

**Figure 5.**
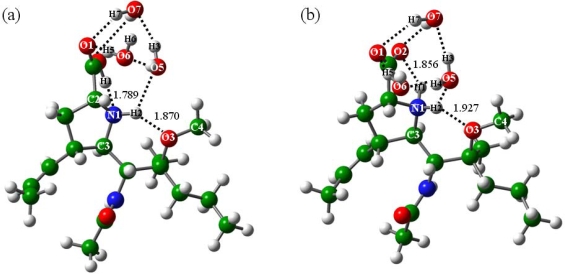
Structures of the BL isomers with addition of three water molecules: (a) BLa3, (b) BLb3.

As shown in Figure 5a, the C1-O2 and C1-O1 distances of BLa3 are close to those of BLa, BLa1 and BLa2, equaling 1.328 and 1.226 Ǻ, respectively. The N1-H1 and O3-H2 H-bonds were optimized at 1.789 and 1.870 Ǻ, respectively. In BLb3, the C1-O2 and C1-O1 distances are equal to 1.262 and 1.253 Ǻ, and the O2-H1 and O3-H2 H-bonds are equal to 1.856 and 1.927 Ǻ, respectively. It indicated that the zwitterions of BL are structurally close in the cases of two and three water molecules. The three water molecules form complex H-bond networks with the BLa and BLb structures [19]. The O5-H2, O1-H5, O1-H7 and O2-H8 H-bonds were optimized at 2.466, 1.902, 2.010 and 2.539 Ǻ in BLa3 as well as 2.005, 1.742, 2.216 and 1.960 Ǻ in BLb3, respectively. That is, the carboxyl-O atoms in both BLa3 and BLb3 are coordination-saturated with three H-bonds of medium strengths, which greatly stabilizes the zwitterionic structure. The three water molecules are connected with each other by the H-bonds, see Figure 5. The energy calculations indicated that with addition of three water molecules (n = 3), the zwitterion of BL (BLb3) is more stable than the corresponding canonical isomer (BLa3), with the energy difference of −1.54 kcal mol^−1^. It is consistent with the results of the above geometric analysis. Compared with the geometries and energies of two and three water molecules, it was found that the increase of the carboxyl-O coordinations by H-bonds may probably the main reason that makes the stability of the zwitterionic BLb3 structure superior to the canonical BLa3 structure, in agreement with the previous results of the zwitterion stabilizations by H-bonds [35–38].

### 2.3. Formation of the Zwitterionic Isomer of BL

**Figure 6.**
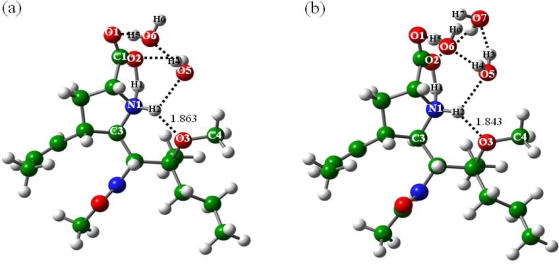
Transition state structures to the BLa and BLb isomers with addition of two and three water molecules: (a) TS2 and (b) TS3.

From the above discussions, it was found that the addition of two molecules (n = 2) renders the zwitterion of BL geometrically stable. The addition of another water molecule (n = 3) further enhances the zwitterion stability and causes it even more stable than the corresponding canonical isomer. The transition state structures of the conversion processes from the canonical isomers to the zwitterions were determined, see TS2 and TS3 in Figure 6. TS2 and TS3 are the transition states in the presence of two and three water molecules, and their characteristic imaginary frequencies fall at 996.8i and 1071.5i cm^−1^, respectively.

It was found that the geometries of the transition states are close to their corresponding energy minima. That is, TS2 resembles BLa2 and BLb2, whereas TS3 resembles BLa3 and BLb3. The main geometric differences lie in the positions the H1 atoms. In TS2 and TS3, the H1 atoms are at the midways of the O2 and N1 atoms instead of forming direct bonds in the energy minima. The O2-H1 and N1-H1 distances were optimized at 1.284 and 1.246 Å in TS2 and 1.231 and 1.299 Å in TS3, respectively.

The energy barriers of the conversion processes from the canonical isomers to the zwitterions of BL were calculated to be 4.96 kcal mol^−1^ in the presence of two water molecules (n = 2) and 3.13 kcal mol^−1^ in the presence of three water molecules (n = 3), respectively. It indicated the zwitterion formation is facile with addition of two molecules and further facilitated by the addition of more water molecules. In addition, the zwitterion formation of BL can be finished in a single step, different from other NA inhibitors such as BA in Scheme 1b [19]. The most important is that three water molecules have rendered the zwitterion of BL more stable than its canonical isomer; *i.e.,* the zwitterion of BL has obviously superior stability in aqueous solutions where the drugs will be assimilated. Accordingly, the designs based on the BL molecule will have great potential applications in the explorations of potent NA inhibitors.

## 3. Computational Methods

All the calculations were carried out with the aid of the Gaussian03 suite of programs [49]. B3LYP density functional [50,51], which combines Becke’s three-parameter hybrid exchange functional (B3) and Lee, Yang, and Parr correlation functional (LYP), were used to optimize the structures of BL, proline as well as the water-interacting systems. The standard 6-31G(d, p) basis set was used throughout. The transition states (TS) were characterized by numerical frequency analyses that each has only one imaginary vibration.

## 4. Conclusions

In this work, density functional calculations were used to study the stability and formation of the zwitterionic isomer of a proline-based neuraminidase inhibitor; *i.e.,* 5-[(1*R*,2*S*)-1-(acetylamino)-2-methoxy-2-methylpentyl]-4-[(1*Z*)-1-propenyl]-(4*S*,5*R*)-d-proline (BL). The gradual addition of water molecules (n = 0, 1, 2, 3) was considered. The main conclusions were summarized below.

In the absence of water, three isomers exist on the potential energy surface (PES) of BL, with the one that can convert to the zwitterion of the lowest energy. Neither of the BL and proline zwitterions represents a local minimum on the PES, which was confirmed by the additional energy-profile calculations with the gradual changes of the N1-H1 distance (Figure 2) and also agrees well with the previous results of proline conformational analysis. The zwitterions of BL and proline were estimated 13.25 and 15.01 kcal mol^−1^ higher in energy than their corresponding canonical isomers. Accordingly, the introduction of functional groups to proline to form BL enhances somewhat the zwitterion stabilities.

The addition of water molecules (n = 1, 2, 3) exerts slight influences on the geometries of the BL isomers. One water molecule (n = 1) is not sufficient to stabilize the zwitterion of BL, which spontaneously transforms to the canonical isomer and agrees with the situation in the absence of water. Two water molecules (n = 2) render the zwitterion of BL geometrically stable; however, the zwitterion is still 3.13 kcal mol^−1^ higher in energy than the corresponding canonical isomer. The addition of another one water molecule (n = 3) further stabilizes the zwitterionic structure and reduces its energy to be even lower than the canonical isomer, with the difference of −1.54 kcal mol^−1^. It indicated that the zwitterions of BL are mainly stabilized by the H-bonds from the water molecules, especially in the case of three water molecules (n = 3) where the carboxyl-O atoms are saturated with three H-bonds of medium strengths.

As to the conversion processes from the canonical isomers to the zwitterions of BL, the energy barriers were calculated to be 4.96 and 3.13 kcal mol^−1^ in the presence of two (n = 2) and three (n = 3) water molecules, respectively. Accordingly, the zwitterion formation is facile to take place with the presence of two molecules and further facilitated by the addition of more water molecules. Besides the facile conversion, there are another two advantages of using BL as the potential NA drug: The zwitterion formation is finished in a single step and three water molecules are sufficient to render the zwitterion more stable than the canonical isomer. Therefore, the future designs of NA inhibitors should pay more attention to the BL-based compounds.
